# A host–guest approach to ratiometric pH sensing using upconversion nanoparticles†

**DOI:** 10.1039/d5na00145e

**Published:** 2025-05-27

**Authors:** Ivo Rosenbusch, Marylyn Setsuko Arai, Fabio Rizzo, Andrea S. S. de Camargo, Bart Jan Ravoo

**Affiliations:** a Center for Soft Nanoscience and Organic Chemistry Institute, University of Münster Busso-Peus-Str. 10 48149 Münster Germany b.j.ravoo@uni-muenster.de fabio.rizzo@unimi.it; b São Carlos Institute of Physics, University of São Paulo Av. Trabalhador Sao Carlense, 400 13566-590 São Carlos Brazil; c Department of Pharmaceutical Sciences (DISFARM), University of Milan via Golgi 19 20133 Milan Italy; d Federal Institute for Materials Research and Testing (BAM) Richard-Willstätter Str. 11 12489 Berlin Germany andrea.camargo@bam.de; e Otto-Schot Institute of Materials Research, Friedrich-Schiller University Lessingstrasse 12 07743 Jena Germany

## Abstract

Herein, we present a pH-sensitive nanoplatform based on Tm^3+^/Yb^3+^ co-doped upconversion nanoparticles (UCNPs) functionalized with β-cyclodextrin (β-CD) and a pH-responsive nitrobenzoxadiazole dye modified with adamantane (NBD-Ad). The host–guest interaction between β-CD on the UCNP surface and Ad on NBD-Ad enables a stable, water-dispersible ratiometric pH sensor. Upon 980 nm excitation, the UCNPs emit in the UV and blue regions, which overlap with the pH-dependent absorption of NBD-Ad. This overlap induces selective quenching of UV and blue emissions *via* inner filter effect (IFE). The red emission (650 nm) remains stable and is used as an internal reference for ratiometric sensing. The sensor shows a ratiometric response (blue/red) over a pH range of 8.0–11.0 with high reproducibility. The nanoplatform demonstrates excellent reusability and selectivity even in the presence of interferents.

## Abstract 2

A new pH nanosensor based on β-NaYF_4_:Yb^3+^/Tm^3+^ upconversion nanoparticles functionalized with cyclodextrin and a pH-sensitive adamantane-modified nitrobenzoxadiazole dye (NBD-Ad).

## Graphical abstract

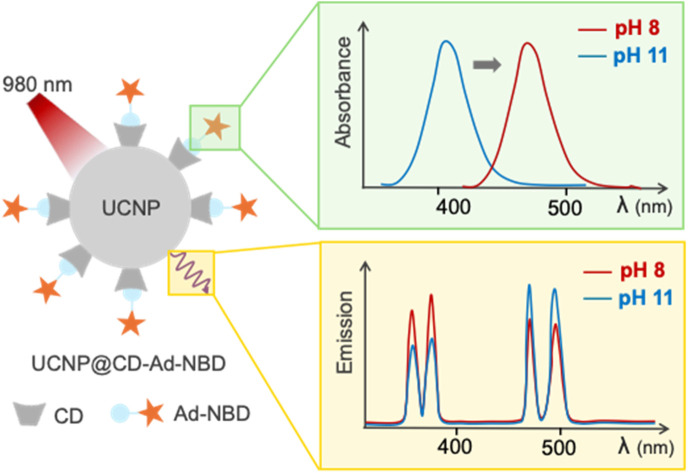

## Introduction

The development of nanoscale pH sensors has gained considerable attention in the past ten years due to their potential to provide precise measurements in confined and complex chemical and biological environments. Conventional pH sensors often fall short when applied to microenvironments such as intracellular spaces, where sensitivity, invasiveness, and the need for minimal sample volumes become critical factors.^1–3^ In this regard, the advent of nanotechnology has opened new avenues for developing sensors that can operate with high sensitivity and precision in such challenging conditions.^4–6^

Upconversion nanoparticles (UCNPs) are among the most promising nanomaterials for sensing applications. UCNPs, typically composed of lanthanide-doped host lattices like NaYF_4_, are unique in their ability to absorb near-infrared (NIR) light and emit at shorter wavelengths through a non-linear process known as upconversion.^7,8^ The process involves the sequential absorption of multiple low-energy photons, which are then emitted as higher-energy radiation in the visible or ultraviolet range, depending on the emitting lanthanide used as dopant ion, (*e.g.*, Er^3+^ in the green and red, Tm^3+^ in the UV, blue and red).^9,10^ In both cases, co-doping with Yb^3+^ is often employed, as it is an efficient sensitizer of the excitation light at 980 nm (a spectral region where high power and relatively low cost diode lasers are available), subsequently transferring the energy to the active emitter. In comparison to other nanoscale luminescent species, UCNPs offer many advantages: they exhibit much higher photostability than quantum dots, lower photobleaching than organic and organometallic dyes, and the IR excitation results in lower or inexistent autofluorescence from biological tissues, which is crucial to obtain high signal-to-noise ratios in biological applications.^11–13^ These properties make them particularly suitable for applications in bioimaging,^14^ sensing,^15–18^ and therapeutic monitoring.^19^

The versatility of UCNPs can be further enhanced by functionalizing their surface with environmentally responsive molecules. Host–guest chemistry, particularly using cyclodextrins (CDs) as host molecules, offers many possibilities to modify nanoparticle surfaces as well to improve their dispersion, stability, and selectivity in sensing or targeting.^20–24^ CDs are cyclic oligosaccharides capable of forming inclusion complexes with a wide range of guest molecules. The hydrophobic interior of the CD cavity binds hydrophobic guest molecules, while the hydrophilic exterior ensures water solubility, making CD-functionalized nanoparticles well-suited for aqueous environments.^25–29^ The integration of UCNPs with host–guest systems, utilizing CD as hosts and adamantane (Ad) as guest molecules, has been investigated in several applications, including bioimaging,^30^ cancer therapy,^31,32^ and molecular sensing,^33,34^ among others.^35^

Recent advancements in upconversion nanotechnology have led to hybrid UCNP-based structures optimized for precise pH sensing. By fine-tuning doping concentrations and surface modifications, researchers have enhanced sensitivity and biocompatibility, enabling real-time, non-invasive monitoring in biological environments.^36^ Notably, polyethylenimine (PEI)-coated UCNPs conjugated with a pH-sensitive indicator have demonstrated great potential for intracellular pH sensing, offering ratiometric luminescence detection over the pH 5 to 7 range with high photostability and minimal autofluorescence.^37^ In this scenario, by exploring the UCNP properties and the versatility of host–guest systems, we present a pH-sensitive nanoplatform based on Tm^3+^/Yb^3+^ co-doped UCNPs functionalized with β-CD and the pH-responsive NBD dye modified with Ad (NBD-Ad). The host–guest interaction between CD on the UCNP surface (UCNP@CD) and Ad on NBD-Ad enables a stable, water-dispersible ratiometric pH sensor. The spectral overlap between NBD-Ad absorption and UCNP emission leads to pH-dependent quenching of the UV and blue luminescence of Tm^3+^, while the red emission remains unaffected and serves as an internal reference. Under basic conditions, the NBD-Ad absorption blue shifts, enhancing UV quenching and partially recovering the blue emission, allowing pH monitoring *via* the blue-to-red (B/R) emission ratio. This strategy not only tunes UCNP luminescence in response to pH, but also demonstrates the versatility of host–guest chemistry for sensor optimization.

The ratiometric sensing mechanism developed in this work enhances accuracy by compensating for variables such as sensor concentration and light source intensity, ensuring highly reliable quantitative analysis. Specifically, the sensor demonstrates excellent performance for monitoring pH changes within the range of 8 to 11. Its effective operation, coupled with the stability afforded by host–guest interactions, positions it as a promising tool for various applications, including intracellular pH monitoring, environmental sensing, and real-time pH regulation in industrial processes.

## Results and discussions

### Ratiometric pH sensor working principle

**Fig. 1:**
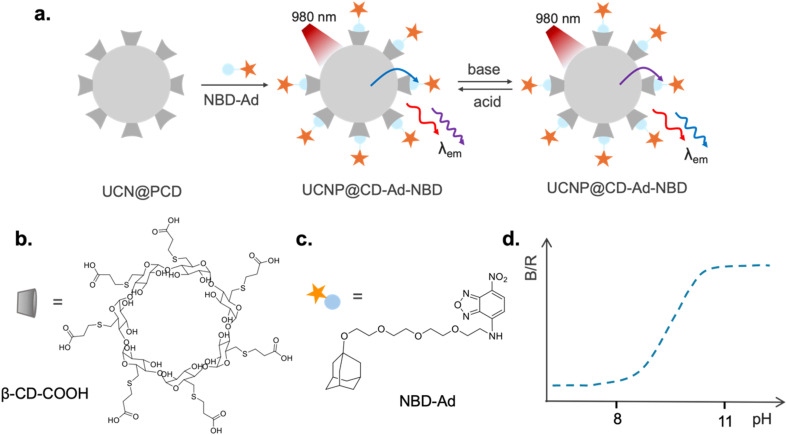
pH sensor working principle. (a) Schematic representation of the produced sensor based on UCNP@CD and NBD-Ad. Molecular structure of (b) CD-COOH and (c) NBD-Ad. (d) UCNP@CD-Ad-NBD B/R emission intensity ratio variations with pH changes.

The developed ratiometric pH sensor, schematically shown in Fig. 1a, is based on the interaction between the NBD-Ad dye and the UCNP@CD (CD = β-CD-COOH, Fig. 1b) to produce the UCNP@CD-Ad-NBD probe. This sensor operates on the principle that the absorption of the NBD-Ad dye (Fig. 1c) correlates with the emission of the nanoparticle, with the UNCP's signal being more quenched either in the UV or blue spectral range depending on the pH of the medium. Under acidic conditions (pH < 6 for the unbonded molecule), NBD-Ad presents an intense absorption band that perfectly matches the Tm^3+^ emissions around 460 nm, so that the blue emissions intensities of the particles are increasingly reduced in the presence of the dye. Conversely, in an alkaline solution (pH > 6), the dye's absorption shifts to shorter wavelengths and overlaps more significantly with Tm^3+^ emissions in the UV. In this way, an increase in the pH causes a concomitant partial recovery of the UCNP's blue emission with a decreased UV emission. The UCNP's red emission remains unaffected, so, following the blue-to-red (B/R) ratio with pH changes (Fig. 1d), it was possible to create a novel ratiometric pH sensor that works in the pH range 8 to 11.

### UCNP@CD synthesis and characterization

**Fig. 2:**
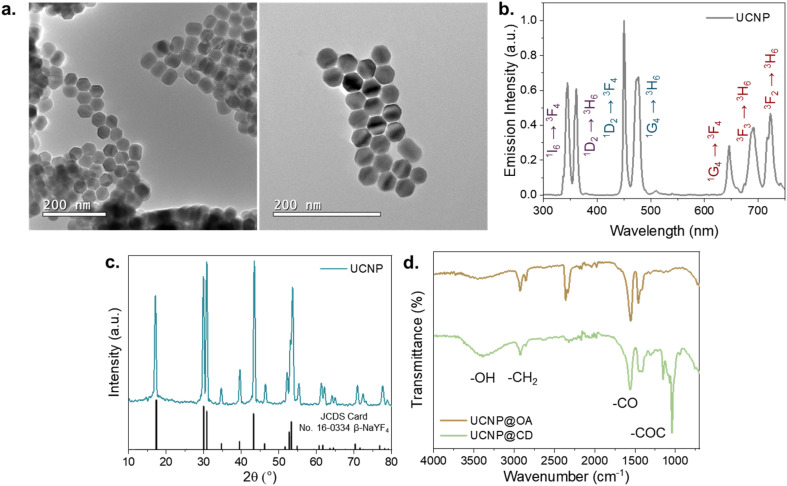
UCNP characterization. (a) Transmission electron microscopy images of the synthetized nanoparticles. (b) Emission spectrum of UCNPs in powder (*λ*_exc_ = 980 nm – 1 W). (c) X-ray diffraction pattern of UCNPs and the calculated pattern of the hexagonal NaYF_4_ matrix. (d) Comparison between the Fourier transform infrared spectra of UCNP@OA and UCNP@CD.

The NaYF_4_:25% Yb^3+^, 0.3% Tm^3+^@NaYF_4_ UNCPs were synthesized through the high-temperature coprecipitation method employing oleic acid (OA) as a chelating agent to give hydrophobic nanoparticles. The obtained UCNPs are highly crystalline, monodisperse, and have an average diameter of approximately 40 nm, as shown in the transmission electron microscopy (TEM) images in Fig. 2a. The particles present a characteristic hexagonal prism shape, which may appear hexagonal when viewed from the top and cubic or rectangular from the side, potentially giving the false impression of morphological heterogeneity. However, all particles belong to the same hexagonal phase, as further confirmed by the X-ray diffraction (XRD) pattern (Fig. 2c), which matches well with the calculated reference (JCPDS No. 16-0334) for pure-phase hexagonal NaYF_4_.

The emission spectrum of UCNPs upon NIR excitation is shown in Fig. 2b. In this system, Yb^3+^ ions act as sensitizers, absorbing the incident photons and transferring the energy to Tm^3+^ active ions, leading to UV-visible light emission. Upon excitation at 980 nm, the Tm^3+^ ions present emissions in the UV at 350 nm and 360 nm, corresponding to the ^1^I_6_ → ^3^F_4_ and ^1^D_2_ → ^3^H_6_ transitions, in the blue at 450 nm and 475 nm, corresponding to the ^1^D_2_ → ^3^F_4_ and ^1^G_4_ → ^3^H_6_, and in the red at 649, 690, and 725 nm corresponding to the ^1^G_4_ → ^3^F_4_, ^3^F_3_ → ^3^H_6_ and, ^3^F_2_ → ^3^H_6_ transitions, respectively.

The addition of β-CD to the UCNP's surface was achieved through a ligand exchange reaction in which CD-COOH replaced the oleic acid on the particle surface. The macrocyclic β-CD ligands, containing seven carboxylic acid functionalities, bind to the UCNPs in a multivalent mode, effectively replacing the weaker, monovalent OA ligands.^38^ After the exchange, the particles became stable in aqueous media, and their zeta potential changed from −13 mV to −40 mV, indicating the successful exchange. Fourier transform infrared absorption (FT-IR) measurements further confirmed the ligand exchange (Fig. 2d). The spectra of UCNP@OA and UCNP@CD were compared, showing that the two bands at 2854 cm^−1^ and 2923 cm^−1^, corresponding to the symmetric and asymmetric –CH_2_ stretches, were present in both samples. A broad peak in the 3500 cm^−1^ region, due to O–H stretching of carboxylic acid groups (–COOH), was more pronounced in UCNP@CD, indicating a higher amount of –COOH groups. Additionally, the appearance of peaks at 1208 cm^−1^ and 1030 cm^−1^ corresponding to C–O and C–O–C stretching vibrations of CD confirmed the ligand exchange. While our methodology was designed as a ligand exchange procedure to replace oleate with carboxylated β-cyclodextrin (β-CD-COOH), we acknowledge that partial self-assembly interactions between residual oleate ligands and CD molecules cannot be fully excluded, especially in cases where complete exchange is not achieved. Nonetheless, the successful attachment of NBD-Ad through Ad/CD host–guest interaction and the stable dispersion of UCNP@CD in water confirm that β-CD-COOH was effectively anchored to the nanoparticle surface to a degree sufficient for our sensing application. In this way, these results demonstrate the successful synthesis and functionalization of stable, water-dispersible UCNP@CD.

### NBD-Ad and its pH-dependent properties

**Fig. 3:**
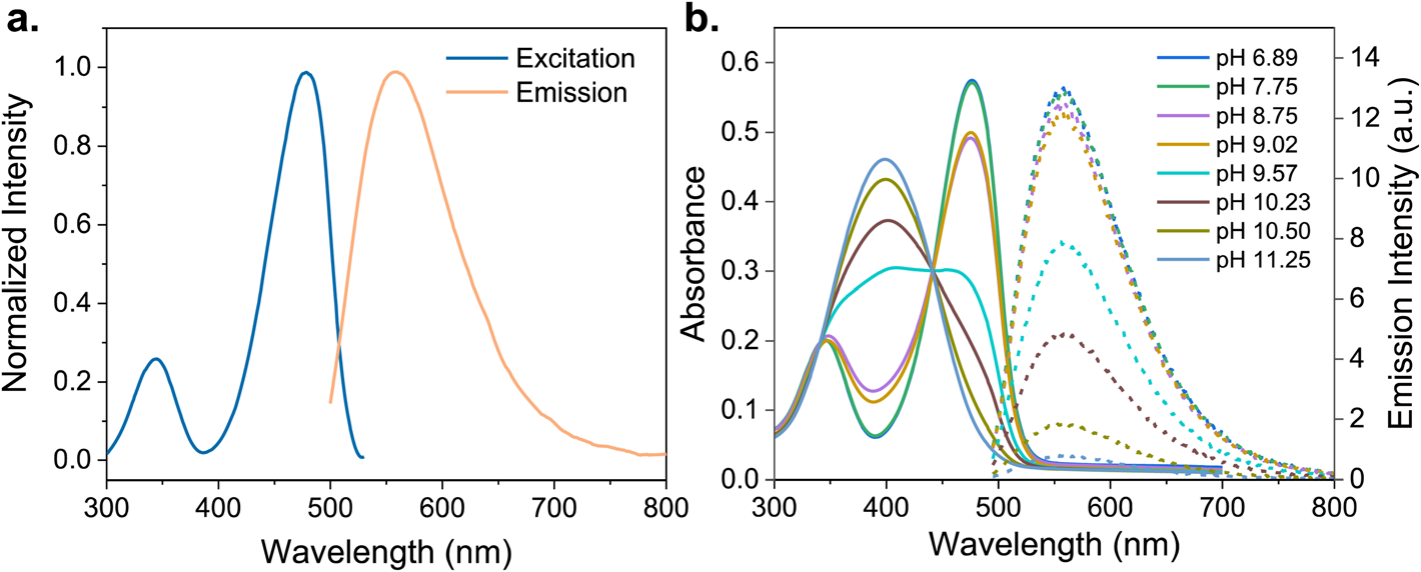
Photophysical properties of NBD-Ad (25 μM) in aqueous solution. (a) Normalized excitation (*λ*_em_ = 580 nm) and emission (*λ*_exc_ = 475 nm) spectra measured at pH 5. (b) Absorption (solid lines, left *y*-axis) and emission (dotted lines, right *y*-axis) spectra recorded at different pH values (pH 3.35–11.25).

NBD-Cl is a hydrophobic nonfluorescent compound that becomes highly fluorescent after reacting with amino groups due to intramolecular charge transfer. Depending on the side chains, water-soluble derivatives can be obtained. To explore the host–guest interactions with CDs in an aqueous environment, we synthesized NBD-Ad through a five-step procedure. Details of the synthesis and analysis of NBD-Ad are available in the ESI.† Adamantane acts as a guest for CD and plays a crucial role in stabilizing the molecular architecture of the dye. The formed NBD-Ad is stable in aqueous solution and exhibits pH-dependent optical properties, with characteristic absorption and emission maxima around 475 nm and 580 nm, respectively, as shown in Fig. 3.

NBD-Ad absorption measurements were conducted in aqueous solutions at different pH values, revealing a spectral shift towards longer wavelengths at lower pH values, as shown in Fig. 3. As the pH decreases from 11.25 to 6.89, the red shift in absorption is approximately 90 nm. This pH-dependent behavior is attributed to the protonation or deprotonation of the amino groups present in the NBD-Ad molecules. Similar phenomena have been observed in analogous NBD derivatives and are attributed to changes in the electron-donating ability of the amino group, which alter the dye's electronic structure and absorption properties.^39,40^

### UCNP@CD complex with NBD-Ad

**Fig. 4:**
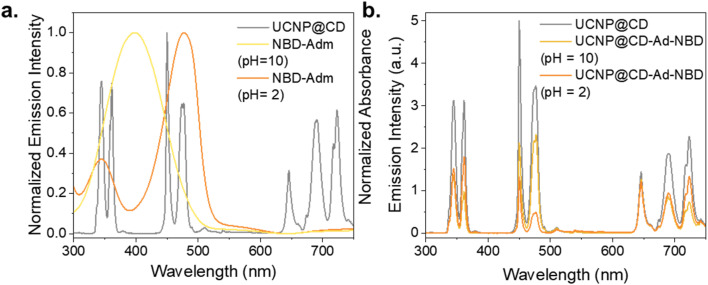
(a) Spectral overlap between the emission of UCNP@CD (0.5 mg mL^−1^, *λ*_exc_ = 980 nm – 1 W) and the absorption of NBD-Ad (50 μM) under acidic and basic conditions. (b) Comparison between the emission of UCNP@CD (0.5 mg mL^−1^, *λ*_exc_ = 980 nm – 1 W) and UCNP@CD-NBD-Ad at pH 2 and 10 (0.5 mg mL^−1^ UCNP@CD + 50 μM NBD-Ad, *λ*_exc_ = 980 nm – 1 W).

Fig. 4 shows the correlation between the NBD-Ad dye and the emission profile of the nanoparticles. Under acidic conditions, NBD-Ad exhibits a strong absorption band superimposed to the Tm^3+^ emission peaks at 452 nm and 476 nm, leading to a more pronounced quenching of the blue emission. In contrast, in an alkaline environment, the dye's absorption band shifts toward shorter wavelengths, resulting in a larger overlap with the Tm^3+^ emissions at 347 nm and 362 nm (Fig. 4a). Therefore, the upconversion luminescence is quenched in either the UV or blue spectral regions depending on the pH of the surrounding medium. In the emission spectra shown in Fig. 4b, it is possible to observe how the UCNP emission is affected by the presence of NBD-Ad at pH 2 and 10. As expected, the emission at 650 nm remains unaffected by NBD-Ad under both acidic and basic conditions, serving as an internal reference. The other two red emissions at 690 and 720 nm exhibit some quenching, which is likely attributed not to specific interactions, but rather to a combination of increasing dilution effects (due to dye solution addition) and inner filter effects, whereas higher concentrations of the dye attenuate both excitation and emission light.

**Fig. 5:**
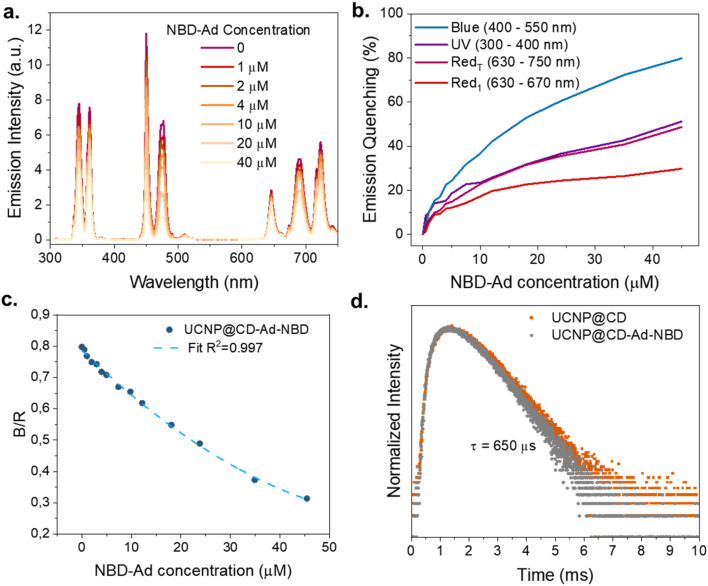
UCNP@CD complex with NBD-Ad (a) emission spectra of UCNP@CD (0.5 mg mL^−1^, *λ*_exc_ = 980 nm – 1 W) with different concentrations of NBD-Ad (0 to 50 μM) at fixed pH = 5.5. (b) Emission quenching (%) of the different emission ranges with increasing NBD-Ad concentration. (c) Change in blue-red ratio with increasing NBD-Ad concentration. (d) Comparison between the 475 nm emission, (*λ*_exc_ = 980 nm – 1 W) lifetime of UCNP@CD (0.5 mg mL^−1^) and UCNP@CD-Ad-NBD.

To build the nanoprobe, UCNP@CD was conjugated with NBD-Ad *via* host–guest interactions to form the UCNP@CD-Ad-NBD platform. Initially, UCNP@CD was mixed with varying concentrations of NBD-Ad to analyze the dye's impact on the nanoparticles' emission (Fig. 5a). These conjugations were realized at pH 5.5, and as the concentration of NBD-Ad increased, continuous quenching in the UCNP emission, especially in the blue region, was detectable. At pH values below 6, the absorption of NBD-Ad significantly overlaps with the UCNP's blue emission, so the pronounced quenching indicates the dye's effect on the nanoparticles' luminescence.

The percentage of emission quenching in different spectral regions (UV: 300–400 nm, Blue: 400–550 nm, Red_1_: 630–670 nm, and Red_T_: 670–750 nm) is shown as a function of NBD-Ad concentration in Fig. 5b. The data highlight that the quenching effect is most pronounced in the blue region (80% at 50 μM NBD-Ad), followed by the UV and Red_T_ spectral regions. A closer inspection of the red emission reveals that the 650 nm band is only slightly affected – showing less than 10% quenching at 5 μM and stabilizing at around 25% above 30 μM – while the emissions at 690 and 720 nm experience more significant attenuation, accounting for the overall red decrease. As previously discussed, this behavior is likely due to a combination of dilution effects and the attenuation of both excitation and emission light at high dye concentrations. In practical sensing applications, however, the concentration of NBD-Ad is expected to be significantly lower, resulting in minimal impact on the red emission. For this reason, the red emissions remain a suitable internal reference, enabling reliable ratiometric measurements. In Fig. 5c, it is possible to observe how the B/R emission ratio decreases with increasing concentrations of NBD-Ad (from 0 μM to 50 μM). The resulting curve fits well with a non-linear regression model, showing a high correlation coefficient (*R*^2^ = 0.997).

The observed quenching in the UCNP@CD-Ad-NBD system can occur through two main mechanisms: Luminescence Resonance Energy Transfer (LRET) and the Inner Filter Effect (IFE), both of which rely on the spectral overlap between the UCNP emission and the dye's absorption. LRET is highly distance-dependent and typically results in a reduction of the donor's emission lifetime, while IFE is governed by the optical density and absorption characteristics of the dye and does not significantly alter the donor lifetime. Despite the strong spectral overlap, fluorescence lifetime measurements of the Tm^3+^ emission at 475 nm (Fig. 5d) showed no significant change – remaining at 650 μs for both UCNP@CD and UCNP@CD-Ad-NBD – indicating that quenching is primarily due to IFE rather than FRET-based mechanisms. This conclusion aligns with the absence of lifetime reduction and supports the role of IFE as the dominant quenching process in our system.

### Host–guest specificity

**Fig. 6:**
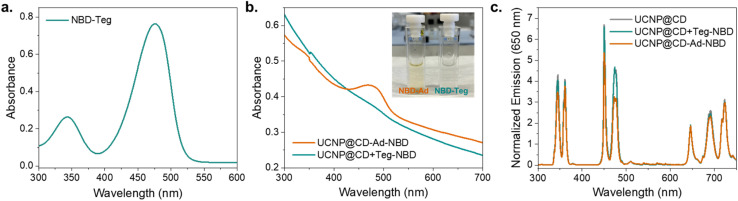
Comparison between UCNP@CD-Ad-NBD and UCNP@CD + Teg-NBD properties. (a) Absorption spectra of NBD-Teg (25 μM) at pH 5. (b) Comparison between the absorption spectra of UCNP@CD-Ad-NBD and UCNP@CD + Teg-NBD (0.5 mg mL^−1^). The inset shows the photograph of cuvettes containing the aqueous solutions (c) normalized emission spectra (at 650 nm) of UCNP@CD, UCNP@CD-Ad-NBD, and UCNP@CD + Teg-NBD (0.5 mg mL^−1^, *λ*_exc_ = 980 nm – 1 W).

To confirm the specificity of the host–guest interaction between Ad and CD on the particles, we conducted a control test using triethylene glycol (Teg) as a substitute for Ad, producing NBD-Teg, which has the same absorbance profile as NBD-Ad as shown in Fig. 6a (see Methods for synthetic details). The UCNP@CD particles were incubated with either NBD-Ad or NBD-Teg. After incubation, the solutions were centrifuged and washed to remove unbound dye molecules. The presence of a host–guest interaction was determined by comparing the binding efficiency of the dyes to the particles. The results shown in Fig. 6b and c demonstrate that NBD-Teg does not bind to CD, ensuring that the observed effects are due to specific host–guest interactions between Ad and CD.

The absorption spectrum of Tm@CD-Ad-NBD (Fig. 6b) exhibits a distinct band around 400 nm corresponding to the absorption of the NBD-Ad, indicating bonding with the particles. In contrast, Tm@CD + Teg-NBD displays a steady absorption curve without any pronounced bands, suggesting that NBD-Teg did not bind to CD. The inset in Fig. 6b displays a photograph of two cuvettes containing the aqueous dispersions of UCNP@CD-Ad-NBD and UCNP@CD + Ad-Teg. The cuvette on the left containing the yellowish solution UCNP@CD-Ad-NBD demonstrates host–guest interaction between Ad on NBD and CD on the particles since the yellow color is attributed to the NBD molecule. Conversely, the cuvette on the right holds the clear, colorless solution UCNP@CD + Teg-NBD, illustrating that Teg does not engage in a host–guest interaction with CD and that the unbound NBD-Teg molecules are removed by the subsequent washing steps. In addition, when comparing the emission spectra (Fig. 6c) of UCNP@CD, UCNP@CD-Ad-NBD, and UCNP@CD + Teg-NBD it can be observed that UCNP@CD-Ad-NBD displays a significant emission decrease at 500 nm. This quenching indicates that NBD-Ad is successfully bound to the particles and filters the UCNP@CD emissions. In contrast, the UCNP@CD + Teg-NBD presented a spectrum very similar to UCNP@CD, with no detected changes in the emissions, indicating the absence of a significant amount of the dye bound at the surface after centrifugation and washing.

Based on these results, we conclude that the specific interaction between Ad and CD facilitates the binding of NBD-Ad to the particles, as evidenced by the distinct yellow coloration, given the absorption at 400 nm, and significant quenching of the UCNP emission around 500 nm. Conversely, the lack of binding with NBD-Teg, demonstrated by the absence of color change, indistinct absorption peaks, and insignificant quenching, confirms the specificity and effectiveness of the host–guest interaction between Ad and CD.

### UCNP@CD-Ad-NBD properties

To evaluate the photophysical interactions between NBD-Ad and UCNP@CD, the studies of the previous sections were conducted by directly mixing increasing concentrations of NBD-Ad with UCNP@CD suspensions. In this setup, excess NBD-Ad remained in solution and likely contributed to nonspecific effects on the UCNPs emission. For the sensing experiments, however, a different preparation protocol was used to ensure that the luminescence changes arose from well-defined host–guest interactions. UCNP@CD particles were incubated with NBD-Ad for one hour, then centrifuged and washed thoroughly to remove any unbound dye. This washing step ensured that only NBD-Ad molecules bound to the β-CD-functionalized particle surface were retained in the final UCNP@CD-Ad-NBD sample. This purified nanoprobe enabled more controlled and reproducible sensing behavior, with minimal background interference from free dye in solution.

The absorption behavior of NBD-Ad remains consistent upon attachment to the particle surface, with the pH-dependent spectral shifts of UCNP@CD-Ad-NBD matching those observed for the free dye, as shown in Fig. 3. The spectra demonstrate the pH-sensitive behavior of UCNP@CD-Ad-NBD nanoprobes by displaying the absorption at pH values of 5.4, 8.3, 9.4, and 10. These spectral shifts correlate directly with protonation changes in NBD-Ad, influenced by adjustments in pH through acid or base addition. The presence of CD on the nanoparticle surface broadens the pH sensitivity range to between 8 and 11.

### pH sensing with UCNP@CD-Ad-NBD

**Fig. 7:**
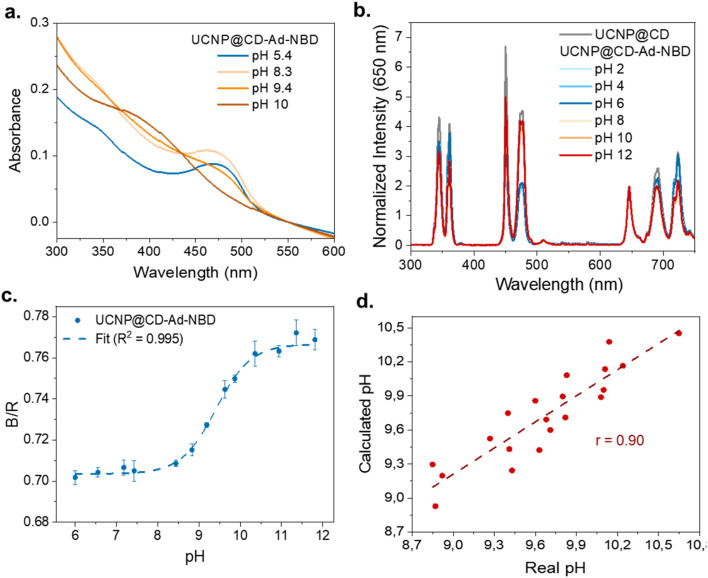
(a) Absorption spectra of UCNP@CD-Ad-NBD nanoprobes at varying pH values (5.4, 8.3, 9.4, and 10). (b) Normalized emission (at 650 nm) of UCNP@CD and UCNP@CD-Ad-NBD for various pH values. (c) Blue-to-red (B/R) emission ratio as a function of pH, with experimental data points and a Boltzmann fit. (d) Correlation between the calculated and real pH values of 18 samples.

The emission spectra of UCNP@CD-Ad-NBD were recorded across a range of pH values and normalized at 650 nm, as shown in Fig. 7b. As expected, the blue emission is more quenched at lower pH values, while the UV emission becomes more quenched at higher pH, consistent with the pH-dependent absorption behavior of NBD-Ad. The integrated areas of the blue (400–500 nm) and red (600–750 nm) emissions were used to calculate the B/R ratios for each pH value, with the resulting relationship shown in Fig. 7c. The data was fitted using a Boltzmann fit, highlighted by the dashed line, representing the calibration curve of the sensor.

At lower pH values (6.0–7.5), the B/R emission ratio remains relatively stable, indicating minimal changes in the emission properties. However, between pH 8 and 11, a pronounced increase in the B/R ratio is observed, demonstrating the sensor's heightened sensitivity to pH changes in this critical range. Beyond pH 11, the B/R ratio stabilizes again, reaching a plateau at higher pH values (up to 12), where the sensor's sensitivity to further changes diminishes. These results confirm that the nanosensor is highly effective for detecting pH variations in the range of 8 to 11. Although NBD-Ad alone exhibits a broader pH-dependent absorption profile when measured by direct absorbance, the sensing behavior of UCNP@CD-Ad-NBD is primarily governed by the interaction between the UCNP emission and the dye's pH-dependent absorption. This interaction defines the effective sensing range of the system. Compared to free NBD-Ad, the slightly narrower response window observed in our nanosensor arises from the selective overlap and energy transfer dynamics between UCNP emission bands and the dye's absorption spectrum. However, this configuration enables a ratiometric and background-free readout, significantly improving the robustness and reliability of the sensor for practical applications.

To validate the practical performance of the nanosensor, its pH sensing capability was tested with 18 aqueous solutions of varying pH values. The pH values determined using UCNP@CD-Ad-NBD were compared against those measured with a laboratory pH meter. The results, presented as a scatter plot in Fig. 7d, show a strong positive correlation between the calculated pH values (*y*-axis) and the real pH values (*x*-axis) measured by the pH meter. The linear regression model demonstrates excellent agreement, with a Pearson correlation coefficient (*r*) of 0.90, underscoring the sensor's accuracy and reliability.

To assess the sensor's robustness in biologically relevant conditions, we evaluated its performance in the presence of common biological interferents, including Mg^2+^ ions and bovine serum albumin (BSA), as well as in phosphate-buffered saline (PBS). The results, shown in Fig. S10,† indicate that the B/R ratio remains stable across all tested conditions, demonstrating the sensor's resistance to interference from these components. This stability in biologically relevant environments suggests that the sensor is well-suited for future applications in physiological settings, where such interferents are present. Additionally, the sensor's cyclability was evaluated by monitoring the B/R ratio during repeated pH cycles between 9 and 10 (Fig. S11†). The response followed the expected trend in the first cycle, with a B/R value of ∼0.72 at pH 8.87. Slight variability appeared in later cycles, likely due to partial dye degradation or changes in host–guest interactions under high pH. Nevertheless, the overall trend remained consistent, confirming the system's reliability for repeated pH sensing and its suitability for practical applications. These findings highlight the sensor's potential for real-world applications in biomedical, environmental, and industrial fields.

## Conclusion

In conclusion, we developed a novel ratiometric pH sensor using β-NaYF_4_:Yb^3+^/Tm^3+^ upconverting nanoparticles functionalized with cyclodextrin (CD) and coupled with a pH-responsive adamantane-modified nitrobenzoxadiazole dye (NBD-Ad). The host–guest interaction between CD and Ad ensured the stability and water dispersibility of the nanosensor, while the pH-sensitive absorption of NBD-Ad enabled precise modulation of the nanoparticle's blue-to-red (B/R) emission ratio in response to pH changes. The sensor demonstrated sensitivity and accuracy within the pH range of 8 to 11. Through practical validation using real samples, the sensor exhibited a strong correlation with conventional pH meter measurements, underscoring its reliability for real-world applications. The robust performance of this nanosensor highlights the potential of combining upconverting nanoparticles with tailored host–guest systems for applications in intracellular pH monitoring, environmental analysis, and industrial process control. Future work may explore further optimization of the sensor for broader pH ranges and its integration into solid matrices to create an optimized reusable platform.

## Materials and methods

### Synthesis of β-UCNPs (NaYF_4_:25% Yb^3+^, 0.3% Tm^3+^)

The UCNPs with the hexagonal structure were synthesized following a high-temperature co-precipitation method previously reported by Gnanasammandhan *et al.*^41^ LnCl_3_ aqueous solutions (0.78 mL YCl_3_ (1 M), 0.25 mL YbCl_3_ (1 M), and 0.30 mL TmCl_3_ (0.01 M)), were transferred to a 100 mL three-necked round-bottom flask and heated to complete water evaporation. Subsequently, the resulting powder was mixed with 6 mL oleic acid and 15 mL octadecene, heated to 150 °C for 30 min under nitrogen or argon atmosphere to form a homogeneous solution, and then cooled down to room temperature. 5 mL of methanol solution containing NaOH (0.1 g) and NH_4_F (0.148 g) were slowly added into the flask and quickly formed solid-state precipitates in the solution. Subsequently, the solution was slowly heated to 110 °C to evaporate methanol, degassed for 10 min, and then heated to 300 °C and maintained for 1 h under an inert atmosphere. After the solution was naturally cooled down, nanocrystals were precipitated with acetone, isolated by centrifugation (6000 rpm, 10 min), and washed once with acetone and twice with ethanol.

### UCNPs coating with a shell of the undoped matrix (NaYF_4_:Yb, Tm@NaYF_4_)

The procedure to coat the nanoparticles with an inert matrix shell is similar to the one used for the synthesis of core UCNPs. First, 1 mL of YCl_3_ aqueous solution (1 M) was transferred to a 100 mL three-necked round-bottom flask and heated until dryness. Then, the resulting powder was mixed with 6 mL oleic acid (OA), and 15 mL 1-octadecene (ODE), and heated to 150 °C for 30 min to form a yellow homogeneous and clear solution. After cooling to room temperature, as-prepared UCNPs (re-dispersed in 15 mL of cyclohexane) were added to the above solution and the mixture was heated to 100 °C. After removing cyclohexane, the synthesis proceeded following the same steps as that of NaYF_4_:Yb, Er/Tm nanoparticles. The final core–shell nanocrystals were washed with acetone one time, with ethanol two times, and dried at room temperature.

### Ligand exchange reaction of oleic acid to β-CD-COOH

To add the CD to the UCNP surface, a ligand exchange reaction was performed following a protocol previously reported.^38^ 20 mg of dried oleic acid-capped UCNPs and 30 mg β-CD were mixed in 10 mL MilliQ water and sonicated for 2 h. Subsequently, the mixture was extracted twice with diethyl ether to remove the OA. The dispersion was centrifuged (10 000 rpm, 10 min), washed once with MilliQ water and the final particles (UCNP@CD) were dried at room temperature.

### Combination of UCNP@CD with NBD-modified dye

To fabricate the UCNP@CD-dye sensing platforms, 2 mL of UCNP@CD (0.5 mg mL^−1^) were mixed with 200 μL of dye solution (500 μM) and the mixture was stirred for 1 h at room temperature. The particles were then isolated (10 000 rpm, 10 min), washed once with MilliQ water, and redispersed in 2 mL of MilliQ water.

### Protocol for determining the pH of an unknown sample using UCNP@CD-Ad-NBD

To determine the pH of an unknown sample, the UCNP@CD-Ad-NBD nanosensor was employed, and its blue-to-red (B/R) emission ratio was measured and compared against a pre-established calibration curve.

#### Measurement

A defined amount of the UCNP@CD-Ad-NBD nanosensor was added to the unknown sample in a quartz cuvette. The sample volume and the nanosensor concentration were ensured to be consistent with those used during the calibration process. The mixture was incubated for 10 minutes at room temperature to allow equilibrium to be reached between the sensor and the sample. Using a fluorescence spectrometer with 980 nm laser excitation, the emission spectrum of the sample was recorded, focusing on the blue (400–500 nm) and red (600–750 nm) emission regions. The areas under the emission peaks in these regions were integrated to calculate the B/R emission ratio.

#### Data analysis

The measured B/R ratio was compared with the pre-established calibration curve fitted using a Boltzmann function. The calibration curve correlated the B/R ratio to pH values within the optimal sensing range of 8 to 11. Using this curve, the pH of the unknown sample was interpolated.

This method provided a precise and reproducible means of determining the pH of samples, leveraging the ratiometric properties of the UCNP@CD-Ad-NBD nanosensor.

## Conflicts of interest

There are no conflicts of interest to declare.

## Supplementary Material

NA-007-D5NA00145E-s001

## Data Availability

The data supporting this article have been included as part of the ESI.†
