# Supplementary material for: A host–guest approach to ratiometric pH sensing using upconversion nanoparticles
Source: Nanoscale Adv. 2025 May 27;7(13):4142–51. doi: 10.1039/d5na00145e (PMC12110347; doi:10.1039/d5na00145e)
Supplement: NA-007-D5NA00145E-s001 [file NA-007-D5NA00145E-s001.pdf]

Supplementary Information (SI) for Nanoscale Advances.
This journal is © The Royal Society of Chemistry 2025

Electronic Supplementary Information (ESI)

A Host-Guest Approach to Ratiometric pH sensing using Upconversion Nanoparticles

Ivo Rosenbusch[1&], Marylyn Setsuko Arai[2&], Fabio Rizzo[1,3*], Andrea S. S. de Camargo[4,5*], and Bart Jan Ravoo[1*]

[1] Center for Soft Nanoscience and Organic Chemistry Institute, University of Münster, 48149 Münster, Germany

[2] São Carlos Institute of Physics, University of São Paulo,13566-590, São Carlos, Brazil

[3] Department of Pharmaceutical Sciences (DISFARM), University of Milan, via Golgi 19, 20133 Milan, Italy

[4] Federal Institute for Materials Research and Testing (BAM), 12489 Berlin, Germany

[5] Otto-Schott Institute of Materials Research, Friedrich-Schiller University, 07743, Jena, Germany

[&] I.R. and M.S.A. contributed equally to this paper.

[*] Corresponding authors

Andrea S. S. de Camargo
andrea.camargo@bam.de

Bart Jan Ravoo
b.j.ravoo@uni-muenster.de

Fabio Rizzo
fabio.rizzo@unimi.it

Synthesis overview of the NBD-Ad dye.

The NBD-Ad dye was prepared in a five-step synthesis, schematically represented in **Fig. 7**. Each step is described in detail in the following sections.

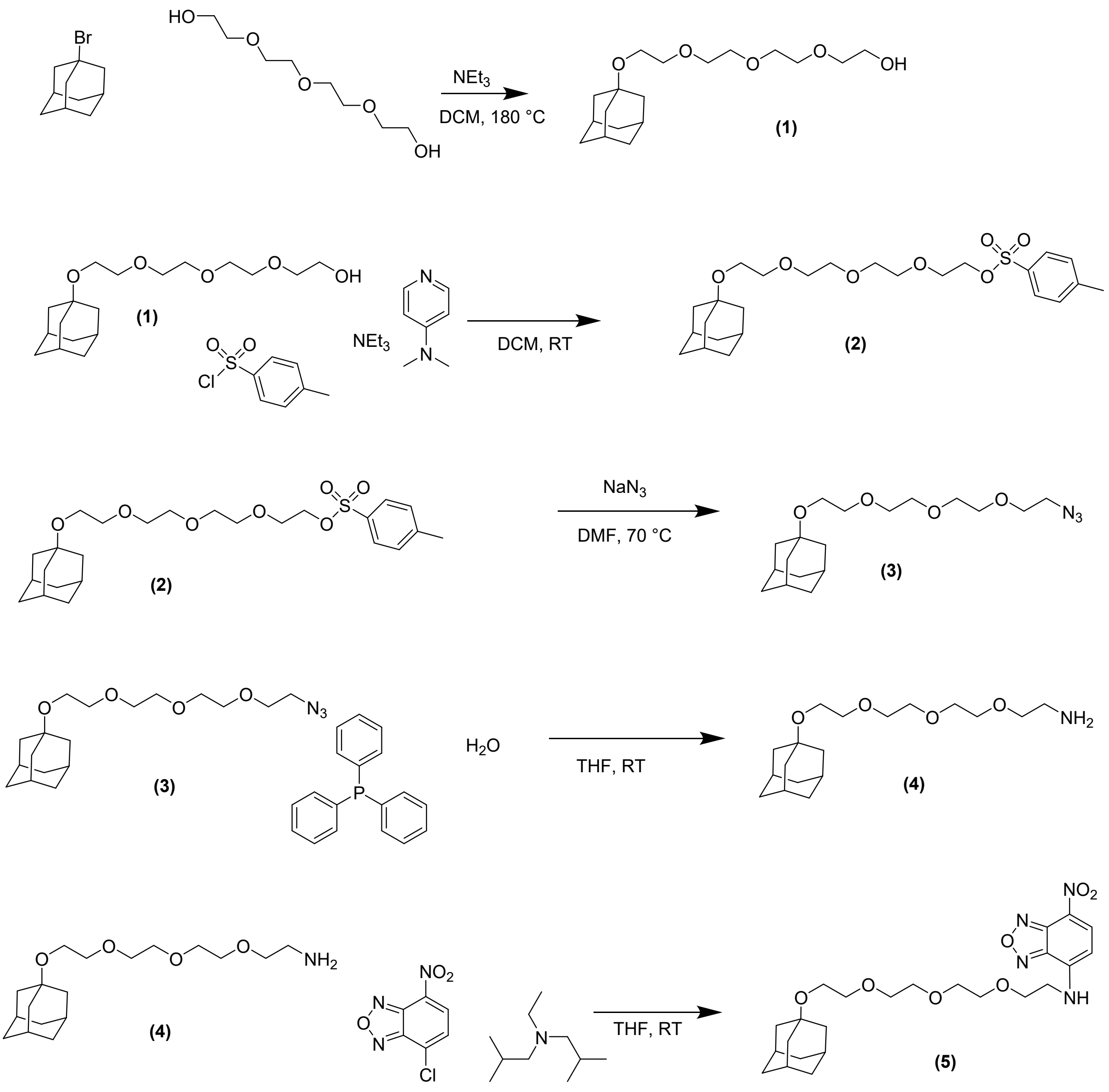

Figure S1. Schematic representation of the NBD-Ad synthesis pathway.

2-(2-(2-(2-(((3s,5s,7s)-adamantan-1-yl)oxy)ethoxy)ethoxy)ethoxy)ethan-1-ol synthesis: (1)

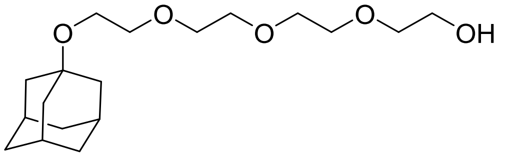

The synthesis was performed according to a known procedure.[35] 1-Bromoadamantane (8 g, 37.2 mmol) and triethylamine (15 mL, 108 mmol) were dissolved in tetraethylene glycol (135 mL). The solution was stirred overnight at 180 °C. It was afterward cooled down to room temperature. 100 mL DCM was added, and the mixture was washed 3 times with 1 M HCL

(50 mL). This was followed by a further wash with brine (50 mL). The combined organic phases were then filtered over $MgSO_4$. The solvent was removed, and the product was obtained as a brown oil. (Yield: 11.75 g, 95%)[1]

2-(2-(2-(2-(((3s,5s,7s)-adamantan-1-yl)oxy)ethoxy)ethoxy)ethoxy)ethyl 4-methylbenzenesulfonate synthesis: (2)

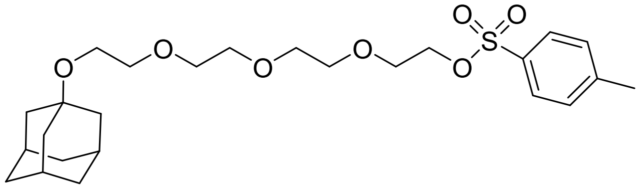

(1) (11.75 g, 35.8 mmol, 1 eq) was dissolved in 100 mL DCM. While stirring, p-toluenesulfonyl chloride (6.82 g, 35.8 mmol, 1eq), Et_3N (5 mL, 35.8 mmol, 1 eq), and DMAP (150 mg, 1.23 mmol, 0.03 eq) were added. After the solution was stirred overnight at room temperature, the reaction was quenched with HCl (1 M, 100 mL). The organic phase was washed twice with water and dried over $MgSO_4$. The solvent was removed under vacuum and column chromatography (cyclohexane/ethyl acetate, 1:1) was carried out for purification. The product was obtained as a yellowish oil. (Yield 9.2 g, 53%)[1]

(3s,5s,7s)-1-(2-(2-(2-(2-azidoethoxy)ethoxy)ethoxy)ethoxy)adamantane: (3)

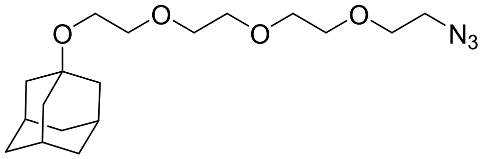

(2) (9.2 g, 19.1 mmol, 1 eq) and NaN_3 (2.48 g, 2 eq) were dissolved in 50 mL DMF under inert conditions. The solution was heated at 70 °C for 18 h with stirring. A mixture of H_2O/DCM (200 mL, 1:1) was then added to the solution. The aqueous phase was separated and washed three times with DCM (50 mL). The combined organic phases were washed with brine and dried over $MgSO_4$. For the final purification, column chromatography was performed. (cyclohexane: ethyl acetate, 1:1). (Yield: 6.4 g, 95%)[2]

^1H NMR (400 MHz, $CDCl_3$) δ 3,62-3.59 (m, 4.5 Hz, 10H), 3.56 – 3.45 (m, 4H), 3.33 (t, J = 5.1 Hz, 2H), 2.08 (dd, J = 6.2, 3.2 Hz, 3H), 1.69 (d, J = 3.1 Hz, 6H), 1.64 – 1.43 (m, 6H).

2-(2-(2-(2-(((3s,5s,7s)-adamantan-1-yl)oxy)ethoxy)ethoxy)ethoxy)ethan-1-amine: (4)

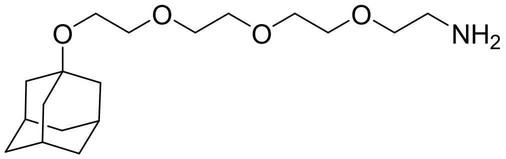

A Staudinger reaction was carried out for the following amine synthesis. **(3)** (6.4 g, 18.1 mmol, 1 eq), PPh_3(5.22 g, 19.9 mmol, 1.1 eq) and H_2O (500 µL, 27 mmol, 1.5 eq.) were dissolved in THF (50 mL). The solution was stirred at room temperature for 3 hours. Subsequently, the solvent was removed under vacuum. The residue was dissolved in water and DCM (100 mL, 1:1). A liquid-liquid extraction was carried out, whereby the aqueous phase was washed three times with DCM. The combined organic phases were dried over $MgSO_4$ and the solvent was removed under vacuum. Finally, the product was purified by column chromatography. (DCM: Methanol, 0.8:0.2). The product was obtained as a colorless oil. (Yield: 4.2 g, 70%)[2]

^{1}H NMR (400 MHz, $CDCl_3$) δ 3.71 – 3.39 (m, 14H), 2.79 (t, *J* = 5.2 Hz, 2H), 2.09 – 2.01 (m, 3H), 1.72 – 1.60 (m, 8H), 1.60 – 1.45 (m, 6H).

N-(2-(2-(2-(2-(((3s,5s,7s)-adamantan-1-yl)oxy)ethoxy)ethoxy)ethoxy)ethyl)-7-nitrobenzo[c][1,2,5]oxadiazol-4-amine: (5)

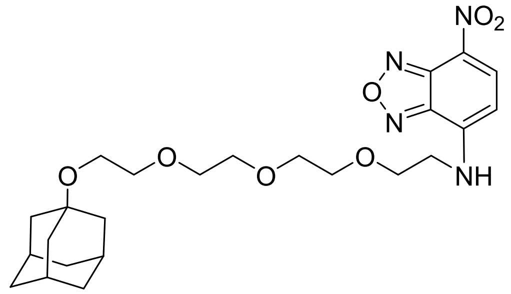

A coupling reaction was carried out to obtain the final product **(5)**. A mixture of **(4)** (300 mg, 916 µmol, 1 eq.) and NBD-Cl (275 mg, 1.4 mmol, 1.5 eq.) was dissolved in 10 mL dry DMF. N-ethyl-N-isobutyl-2-methylpropan-1-amine (0.58 mL, 2.75 mmol, 3 eq.) was added to the reaction mixture. The solution was stirred for 1.5 hours at room temperature and then the solvent was removed in a vacuum. For further purification, column chromatography was carried out using cyclohexane and ethyl acetate as solvents. (gradient 1:0 - 1:1). The final product **(5)** was obtained as a red viscous oil. (Yield: 400 mg, 89%)

^{1}H NMR (400 MHz, $CDCl_3$) δ 8.47 (d, *J* = 8.7 Hz, 1H), 7.80 – 7.53 (m, 1H), 6.19 (s, 1H), 3.88 (dd, *J* = 5.6, 4.3 Hz, 2H), 3.80 – 3.66 (m, 10H), 3.64 – 3.52 (m, 4H), 2.11 (p, *J* = 2.9 Hz, 3H), 1.72 (d, *J* = 2.9 Hz, 6H), 1.70 – 1.47 (m, 6H).

^{13}C NMR (101 MHz, $CDCl_3$) δ 144.47, 136.53, 72.70, 71.57, 70.81, 70.77, 70.67, 70.65, 68.41, 59.38, 41.58, 36.59, 36.55, 30.62.

MS-ESI (m/z): Calculated for [C24H34N4O7Na] + = 513. 23197; Found = 513.23205.

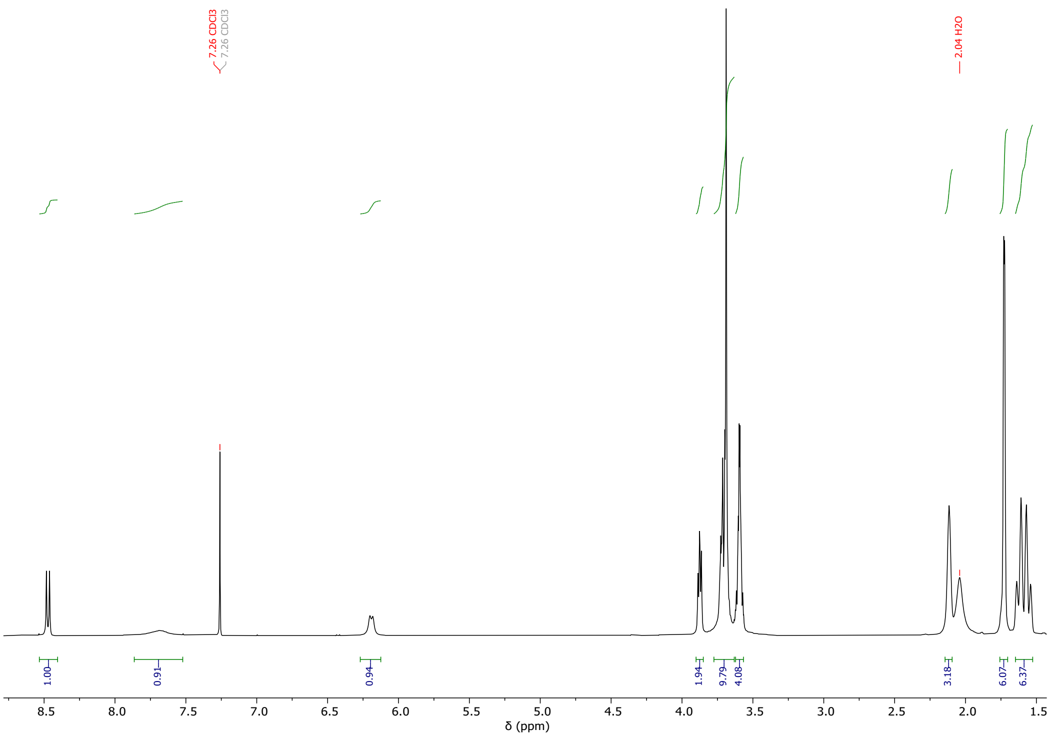

Figure S2. 1H-NMR spectrum of N-(2-(2-(2-(2-(((3S,5S,7S)-adamantan-1-yl)oxy)ethoxy)ethoxy)ethoxy)ethyl)-7-nitrobenzo[c][1,2,5]oxadiazol-4-amine **(5)** recorded in $CDCl_3$at 400 MHz.

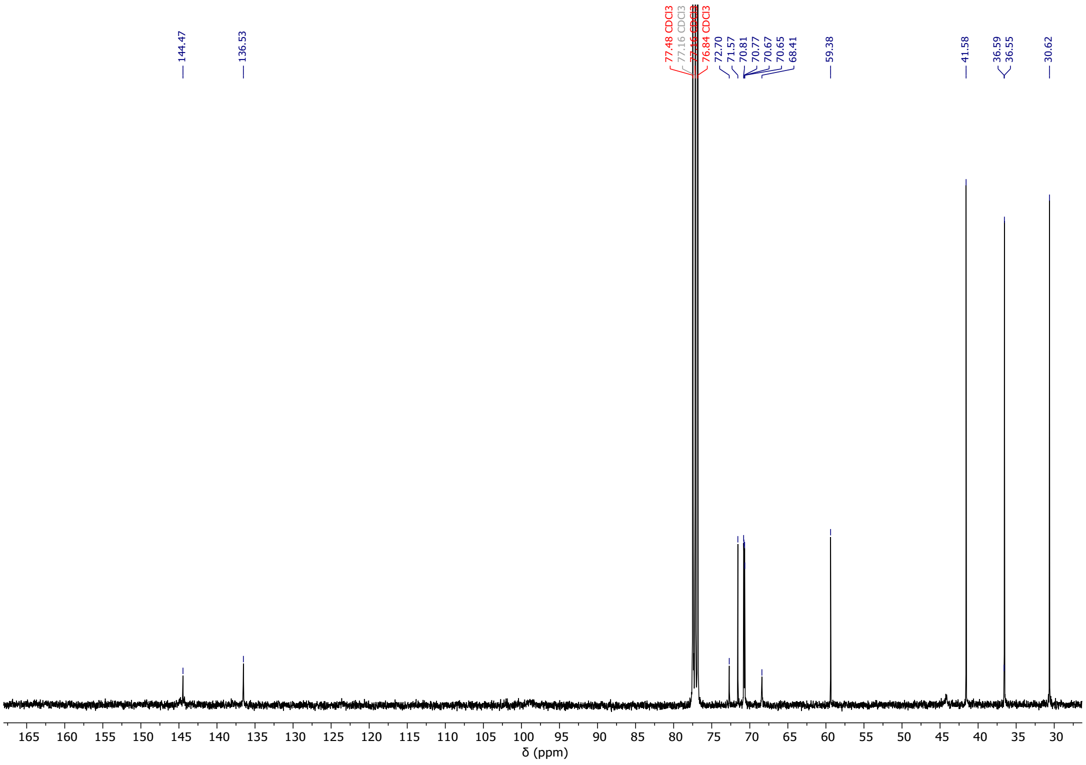

Figure S3. 13C-NMR spectrum of N-(2-(2-(2-(2-(((3S,5S,7S)-adamantan-1-yl)oxy)ethoxy)ethoxy)ethoxy)ethyl)-7-nitrobenzo[c][1,2,5]oxadiazol-4-amine **(5)** recorded in CDCl3_33 at 101 MHz.

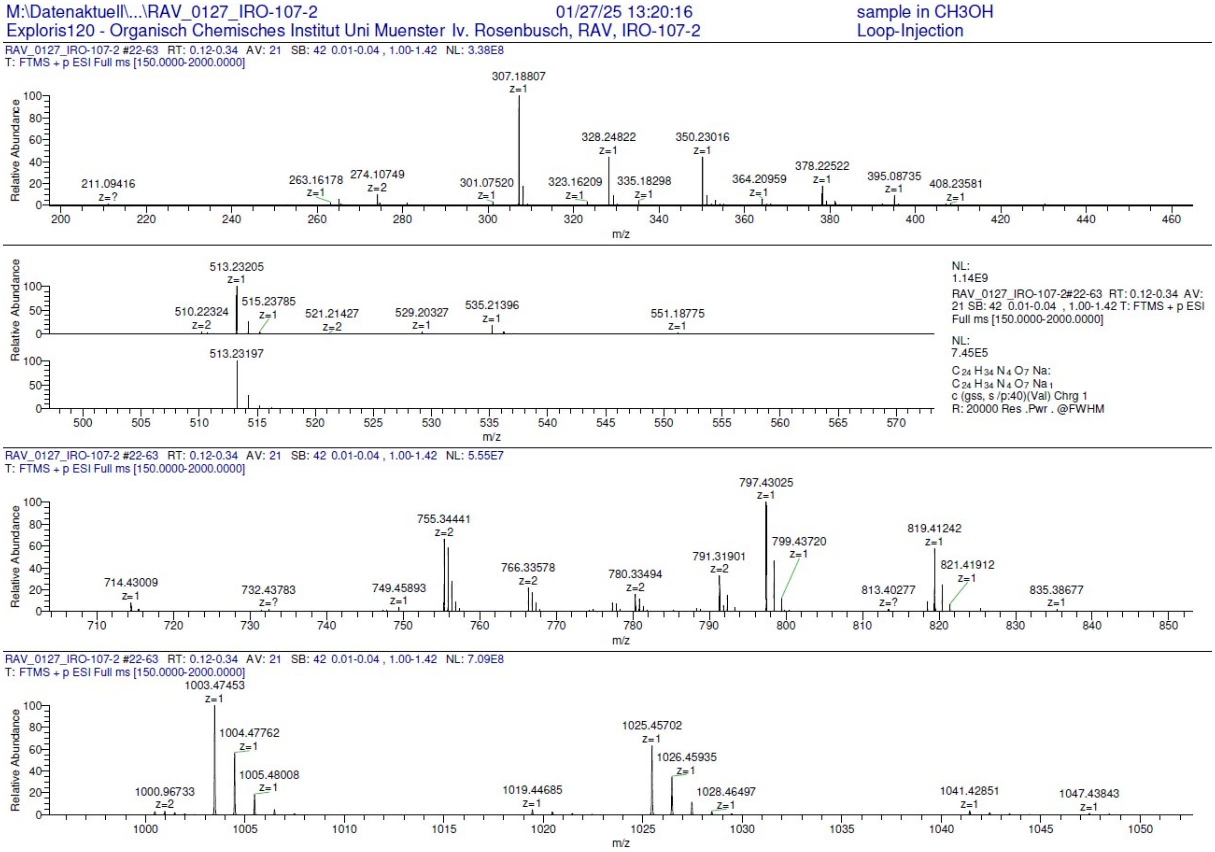

Figure S4. ESI-MS spectrum of N-(2-(2-(2-(2-(((3S,5S,7S)-adamantan-1-yl)oxy)ethoxy)ethoxy)ethoxy)ethyl)-7-nitrobenzo[c][1,2,5]oxadiazol-4-amine **(5)**.

Synthesis of the dye NBD-TEG.

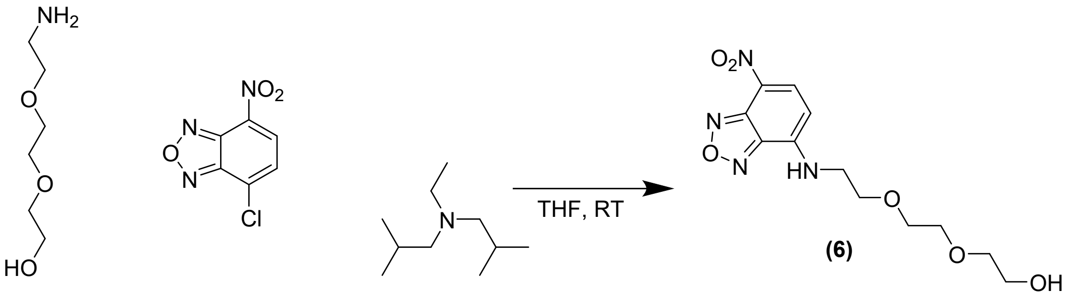

Figure S5. Schematic representation of the synthesis pathway of 2-(2-(2-((7-nitrobenzo[c][1,2,5]oxadiazol-4-yl)amino)ethoxy)ethoxy)ethan-1-ol **(6)**.

2-(2-(2-((7-nitrobenzo[c][1,2,5]oxadiazol-4-yl)amino)ethoxy)ethoxy)ethan-1-ol: (6)

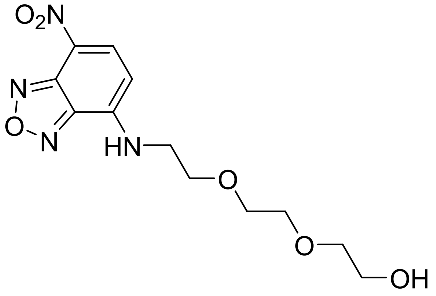

2-(2-(2-aminoethoxy)ethoxy)ethan-1-ol (500 mg, 3.35 mmol, 1 eq.) and NBD-Cl(1 g, 5.03 mmol, 1.5 eq.) were dissolved in dry THF (50 mL). The Hünig base (1.58 g, 10.05 mmol, 3

eq.)was added dropwise over a period of 3 minutes while stirring. The mixture was stirred for 1.5 h at room temperature. The solvent was then removed using a rotary evaporator and the supernatant was purified by column chromatography. (Cyclohexane:ethyl acetate 1:1) The product was obtained as a red solid.

Yield 0,501 g, 50 %

^{1}H NMR (400 MHz, $CDCl_3$) δ 8.49 (d, *J* = 8.6 Hz, 1H), 7.06 (s, 1H), 6.18 (d, *J* = 8.6 Hz, 1H), 3.88 (t, *J* = 5.0 Hz, 2H), 3.81 (t, *J* = 4.5 Hz, 2H), 3.77 – 3.64 (m, 9H).

MS-ESI (m/z): 335.27 ([M+Na]$^+$), found 335.09 ([M+Na]$^+$);

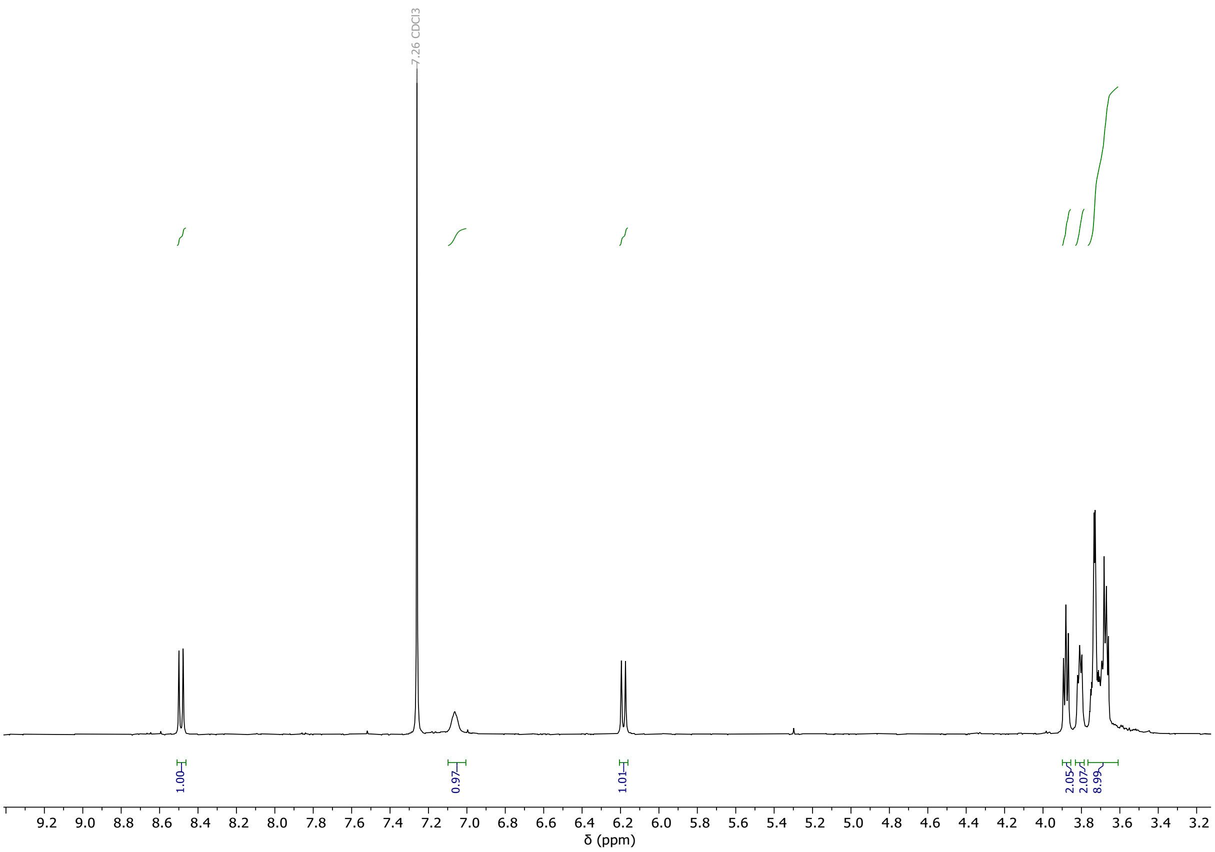

Figure S6. 1H-NMR spectrum of 2-(2-(2-((7-nitrobenzo[c][1,2,5]oxadiazol-4-yl)amino)ethoxy)ethoxy)ethan-1-ol **(6)** recorded in $CDCl_3$at 400 MHz.

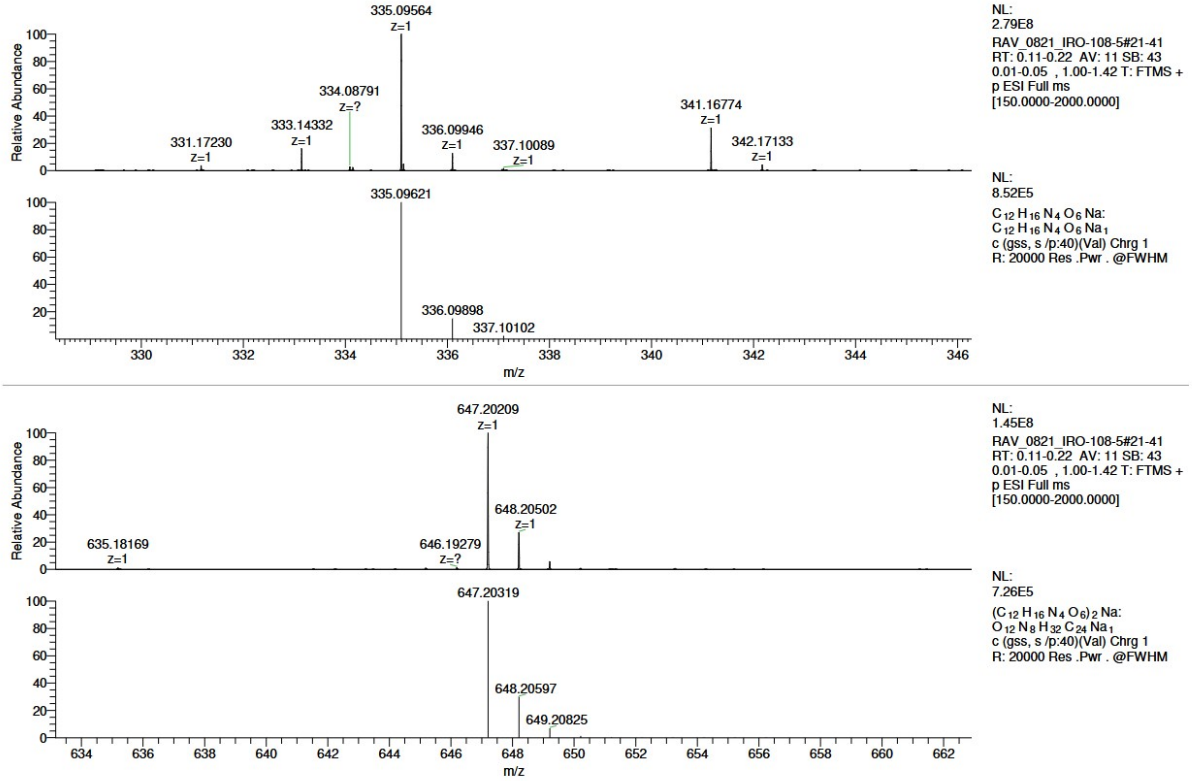

Figure S7. ESI-MS spectrum of 2-(2-(2-((7-nitrobenzo[c][1,2,5]oxadiazol-4-yl)amino)ethoxy)ethoxy)ethan-1-ol **(6)**.

Synthesis overview of β-CD-COOH.

The two synthesis steps of CD-COOH are schematically shown in **Figure S5** and are described in more detail in the following sections.

Synthesis overview of β-CD-COOH. The two synthesis steps of β-CD-COOH are schematically shown in **Figure S5** and are described in more detail in the following sections. This synthesis has been previously reported by Ravoo et al., ensuring its reproducibility and reliability. [3–5]

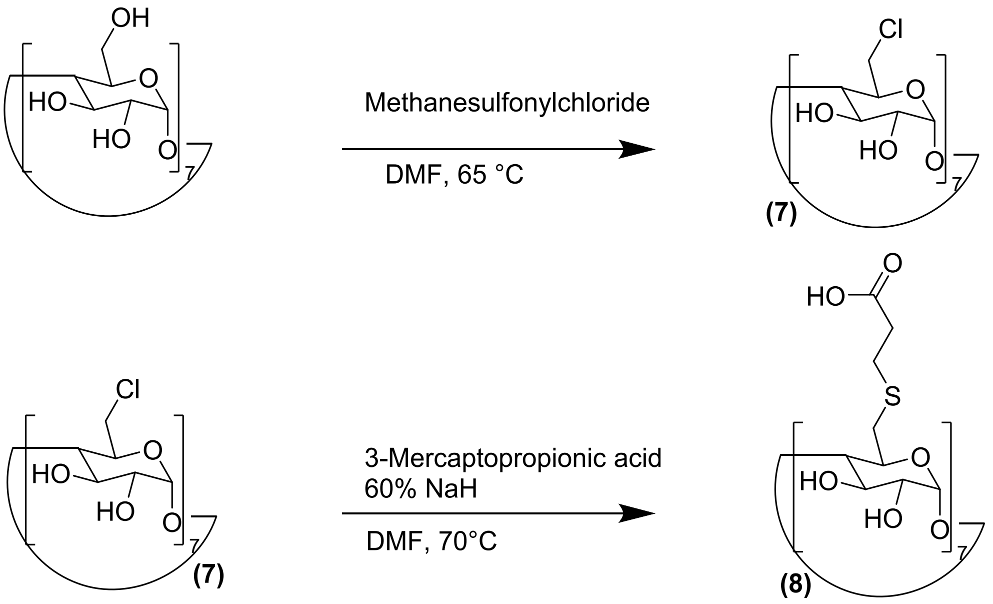

Figure S8. Schematic diagram of the β-CD-COOH synthesis.

Hexachloro-hexadeoxy-β-cyclodextrin (β-CD-Cl): (7)

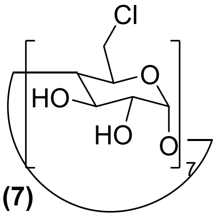

(7)

Following a previously reported work, the β-CD (4.6 g, 4.1 mmol, 1 eq) was dissolved in 70 mL dry DMF. Then methanesulphonyl chloride (13.8 mL, 179 mmol, 44 eq) was added. The solution was heated to 65 °C and stirred for 2 d. After 2 d was cooled to room temperature, and the solvent was evaporated under vacuum. The product was dissolved in methanol and the pH was adjusted to 7. The product was precipitated by the addition of ice water, the solid was filtered and filtered with filtered and washed with MeOH. The product **(7)** was obtained as a white solid. (Yield 6.5 g, 80%)[5]

Per-6-deoxy(carboxylpropyl)thio- β-cyclodextrin: (8)

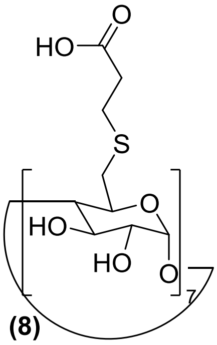

(8)

3-mercaptopropionic acid (7.23 mL, 92 mmol, 35 eq) was dissolved in 90 mL dry DMF. Then, 60% NaH (4.4 g, 203 mmol, 77 eq.) was carefully added and stirred for 90 min. The solution was cooled to 0 °C and **(7)** (3 g, 2.6 mmol, 1 eq) was added in one portion. The solution was heated to 70 °C and stirred for 2 d. At the end of 2 d, it was allowed to cool to room temperature and the solvent was evaporated under reduced pressure. The resulting product was then

precipitated with a large amount of ice-cooled abs. EtOH was precipitated, filtered and dissolved in as little distilled water as possible. The pH was adjusted to 6 and the aqueous solution was dialysed (MWCO 1000) for 3 days, changing the external distilled water from time to time to reduce the excess thiol. At the end of 3 d, the internal solution was freeze-dried and the white solid was washed by Soxhlet extraction with cyclohexane to remove the mineral oil. The purified solid was dissolved in a minimal amount of distilled water, the pH was adjusted to pH 7 by adding a small amount of sodium methoxide and the solution was freeze-dried again. The final product **(8)** was obtained as a white solid. (Yield 1.4 g, 33%).[5]

FTMS (m/z): Calculated for [C63H98O42S7] -3 = 582.44531; Found = 582.44488.

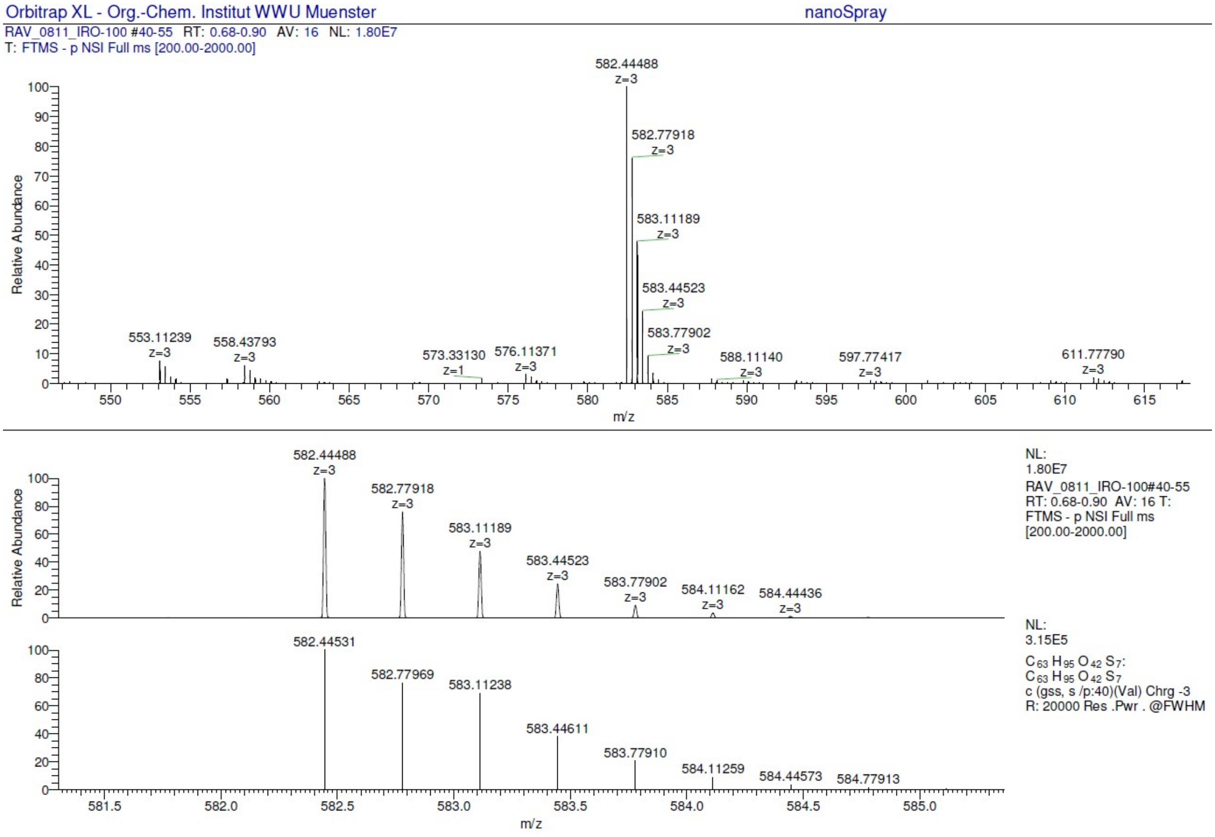

Figure S9. MS spectrum of Per-6-deoxy(carboxylpropyl)thio-β-cyclodextrin (8) recorded using FTMS with nanoSpray ionization.

Quenching percentage calculation

To calculate the quenching percentage (Q%), the recorded spectra were divided into three spectral regions: UV (300–400 nm), blue-green (400–550 nm), and red (600–750 nm). The integrated emission area in the absence of dye was considered as the reference (100% emission, corresponding to 0% quenching). Emission values in the presence of different dye concentrations were then compared to this reference. The quenching percentage was calculated using the following equation: $\mathbf{Q\% = 100 - [(I_2 \times 100) / I_1]}$, where I_1 is the integrated emission without dye and I_2 is the integrated emission with dye.

Calculation of B/R Ratios

To evaluate the pH-dependent ratiometric response of the UCNP-based system, the emission spectra were divided into three spectral regions: UV (300–400 nm), blue-green (400–550 nm), and red (600–750 nm). The B/R ratio was calculated by integrating the emission intensity within the blue-green region (400–550 nm) and dividing it by the integrated intensity in the red region (600–750 nm). This ratio reflects the relative contribution of the two emission bands and enables quantitative comparison across different pH conditions.

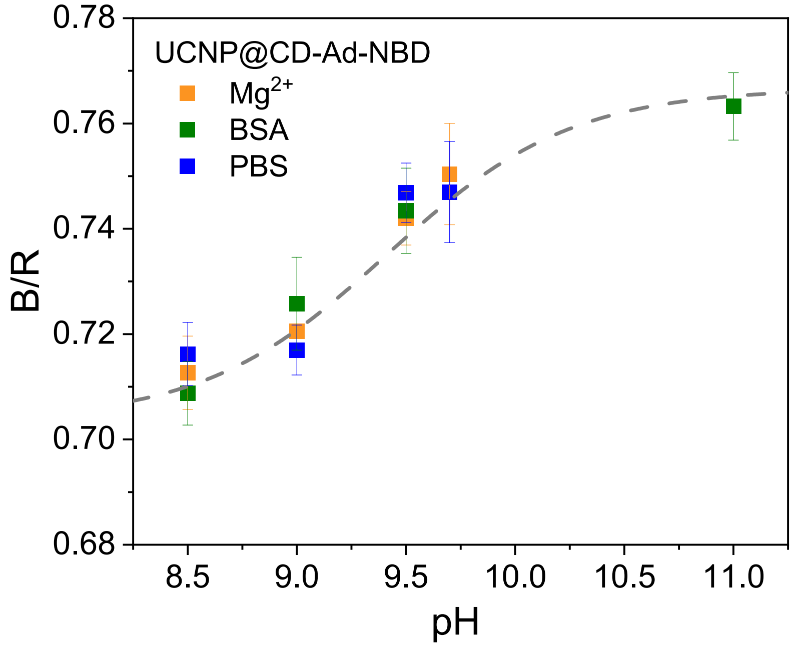

Figure S10. Stability of the sensor's B/R ratio in biologically relevant conditions. The plot compares the response of the sensor (represented by the colored data points) to the calibration curve (dashed line) in the presence of potential biological interferents, including Mg^{2+} ions and bovine serum albumin (BSA), as well as in PBS buffer. The concentration of the UCNP@CD-Ad-NBD nanoprobe used in all experiments was 0.5 mg/mL.

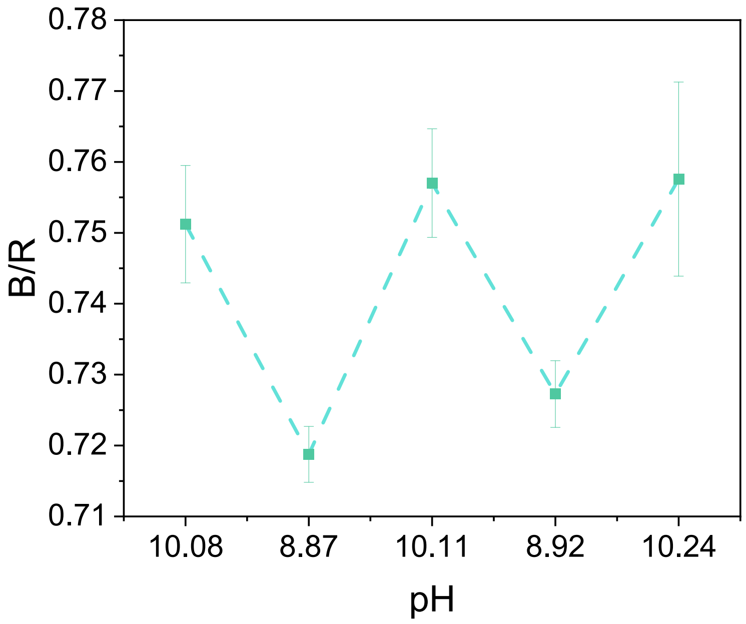

Figure S11. The cyclic response of the sensor showing the reproducibility of B/R ratio at varying pH levels (n=3).

References

1 U. Kauscher and B. J. Ravoo, Mannose-decorated cyclodextrin vesicles: The interplay of multivalency and surface density in lectin-carbohydrate recognition, *Beilstein J. Org. Chem.*, 2012, **8**, 1543–1551.

2 J. L. Gustafson, T. K. Neklesa, C. S. Cox, A. G. Roth, D. L. Buckley, H. S. Tae, T. B. Sundberg, D. B. Stagg, J. Hines, D. P. McDonnell, J. D. Norris and C. M. Crews, Small-Molecule-Mediated Degradation of the Androgen Receptor through Hydrophobic Tagging, *Angewandte Chemie*, 2015, **127**, 9795–9798.

3 S. Engel, N. Möller and B. J. Ravoo, Stimulus-Responsive Assembly of Nanoparticles using Host-Guest Interactions of Cyclodextrins, *Chemistry – A European Journal*, 2018, **24**, 4741–4748.

4 S. Engel, N. Möller, L. Stricker, M. Peterlechner and B. J. Ravoo, A Modular System for the Design of Stimuli-Responsive Multifunctional Nanoparticle Aggregates by Use of Host-Guest Chemistry, *Small*, 2018, **14**, e1704287.

5 N. Möller, T. Hellwig, L. Stricker, S. Engel, C. Fallnich and B. J. Ravoo, Near-infrared photoswitching of cyclodextrin-guest complexes using lanthanide-doped LiYF4 upconversion nanoparticles, *Chem. Commun.*, 2016, **53**, 240–243.
